# Hepatocyte and Sertoli Cell Aquaporins, Recent Advances and Research Trends

**DOI:** 10.3390/ijms17071096

**Published:** 2016-07-09

**Authors:** Raquel L. Bernardino, Raul A. Marinelli, Anna Maggio, Patrizia Gena, Ilaria Cataldo, Marco G. Alves, Maria Svelto, Pedro F. Oliveira, Giuseppe Calamita

**Affiliations:** 1 Department of Microscopy, Laboratory of Cell Biology, Institute of Biomedical Sciences Abel Salazar (ICBAS) and Unit for Multidisciplinary Research in Biomedicine (UMIB), University of Porto, 4050-313 Porto, Portugal; raquellbernardino@gmail.com; 2 Instituto de Fisiología Experimental-CONICET, Facultad de Ciencias Bioquímicas y Farmacéuticas-Universidad Nacional de Rosario, 531 S2002LRK Rosario, Santa Fe, Argentina; rmarinel@unr.edu.ar; 3 Department of Biosciences, Biotechnologies and Biopharnaceutics, University of Bari “*Aldo Moro*”, 70125 Bari, Italy; anna.maggio1988@gmail.com (A.M.); annapatrizia.gena@uniba.it (P.G.); ilaria.cataldo@uniba.it (I.C.); maria.svelto@uniba.it (M.S.); 4 CICS-UBI, Health Sciences Research Centre, University of Beira Interior, 6201-506 Covilhã, Portugal; alvesmarc@gmail.com; 5 Instituto de Investigação e Inovação em Saúde, Universidade do Porto, 4200-135 Porto, Portugal

**Keywords:** mitochondria, reactive oxygen species (ROS), metabolic homeostasis, bile formation, Non-Alcoholic Fatty Liver Disease (NAFLD), male fertility, spermatogenesis, testis, liver

## Abstract

Aquaporins (AQPs) are proteinaceous channels widespread in nature where they allow facilitated permeation of water and uncharged through cellular membranes. AQPs play a number of important roles in both health and disease. This review focuses on the most recent advances and research trends regarding the expression and modulation, as well as physiological and pathophysiological functions of AQPs in hepatocytes and Sertoli cells (SCs). Besides their involvement in bile formation, hepatocyte AQPs are involved in maintaining energy balance acting in hepatic gluconeogenesis and lipid metabolism, and in critical processes such as ammonia detoxification and mitochondrial output of hydrogen peroxide. Roles are played in clinical disorders including fatty liver disease, diabetes, obesity, cholestasis, hepatic cirrhosis and hepatocarcinoma. In the seminiferous tubules, particularly in SCs, AQPs are also widely expressed and seem to be implicated in the various stages of spermatogenesis. Like in hepatocytes, AQPs may be involved in maintaining energy homeostasis in these cells and have a major role in the metabolic cooperation established in the testicular tissue. Altogether, this information represents the mainstay of current and future investigation in an expanding field.

## 1. Introduction

Aquaporins (AQPs) represent channel proteins permeating water, small solutes and certain gases across biological membranes [1]. Mammals have 13 homologues (AQP0–12) playing a number of roles. AQP genes are found in all kingdoms, which highlights their functional significance in living cells. The 2003 Nobel Prize for Chemistry was awarded to Peter Agre for the discovery and functional demonstration [2] of the AQP family of membrane channels (for a letter about Peter Agre, see [3]). Based on their biophysical properties of transport and phylogenesis, mammalian AQPs are grouped into orthodox aquaporins, AQPs primarily permeable to water (AQP0, AQP1, AQP2, AQP4, AQP5, AQP6 and AQP8) and aquaglyceroporins, AQPs transporting a series of small uncharged solutes, particularly glycerol, in addition to water (AQP3, AQP7, AQP9, AQP10). AQP11 and AQP12, two AQPs whose conducting properties are unclear, are considered unorthodox homologues due to their distinct evolutionary pathway. Due to its marked ability to transport ammonia and hydrogen peroxide (besides to water), AQP8 is also indicated as ammoniaporin or peroxiporin.

The expression, biological significance and translational value of AQPs have been the object of intense investigation in all body districts. Unanticipated roles are being found for this family of channels besides more predictable functions. Here, we attempt an overview comprising the most recent advances and research trends on the regulation and function of AQPs in two critically important cell types, namely hepatocytes and Sertoli cells, both in health and disease.

## 2. Hepatocyte Aquaporins: Physiology, Pathophysiology and Potential Relevance as Drug Targets

### 2.1. Expression and Subcellular Localization of Hepatocyte Aquaporins

Liver expresses multiple AQP homologues in virtually all cell types composing the organ, attesting the biological importance of this family of membrane channels (for review, see [4]). Rodent and human hepatocytes express AQP9 and AQP8 at the protein level [4] (for review, see [5]). Hepatocyte AQP8 in its *N*-glycosylated form is found in canalicular membranes and pericanalicular vesicles [6,7,8], while as a non-glycosylated protein is present in inner mitochondrial membranes [9,10]. Hepatocytes are able to hormonally regulate their AQP8-mediated canalicular water permeability. Glucagon induces AQP8 vesicle trafficking to canalicular lipid raft microdomains which is accompanied by an increase in membrane water permeability [11,12,13], via cAMP-protein kinase A (PKA) and phosphatidylinositol 3-kinase (PI3K) signaling pathways [11] and, likely, microtubule-associated proteins [11,14]. Glucagon is also able to upregulate AQP8 protein expression through cAMP-PKA and PI3K pathways [15]. Hence, apical AQP8 would be able to modulate water permeability and facilitate osmotically-driven canalicular water secretion [5,16]. Nevertheless, mitochondrial AQP8 would not play a major role in mediating water transport across mitochondrial membranes [17,18,19]. Based on AQP8 permeabilities to ammonia [20] and hydrogen peroxide [21], recent experimental evidence suggests its participation in mitochondrial ammonia detoxification via ureagenesis [22] and in hydrogen peroxide-mediated cell signaling [23,24].

AQP9 is specifically localized at the hepatocyte sinusoidal membrane [25,26]. In the rodent hepatic lobule, AQP9 displays a heterogeneous expression pattern. Especially in females, hepatocyte AQP9 protein expression is higher in the central vein area by gradually declining towards the periportal area. The extent of expression is also sexually dimorphic [27], as female rats have significantly lower levels of hepatic AQP9 protein compared with males [28,29]. As described below, hepatocyte AQP9 is the main pathway of glycerol uptake [30,31] and urea extrusion [32]. Hepatocyte AQP9 is thought to facilitate the sinusoidal uptake of water with minimal osmotic perturbation. Its expression or localization in hepatocytes does not appear to be modulated by glucagon [11,15]. AQP9 also allows permeation of metalloids such as arsenite, thus providing a route for excretion by the liver [33]. AQP9 has also been described to facilitate the membrane movement of hydrogen peroxide in mammalian cells [34]. Mouse hepatocytes also express AQP11, a homologue implicated in rough endoplasmic reticulum (RER) homeostasis and liver regeneration [35]. Rodent and/or human hepatocytes have also been reported to express other AQPs. However, their actual presence remains arguable as their expression is limited to the transcript or has not been confirmed by all authors addressing the question. Localization and suggested physiological and pathophysiological significance of AQPs in hepatocytes are shown in Table 1.

### 2.2. Involvement in Metabolic Homeostasis and Energy Balance

#### 2.2.1. Aquaporin 9 (AQP9)-Mediated Glycerol Import in Gluconeogenesis and Triacylglycerols Synthesis

**Figure 1:**
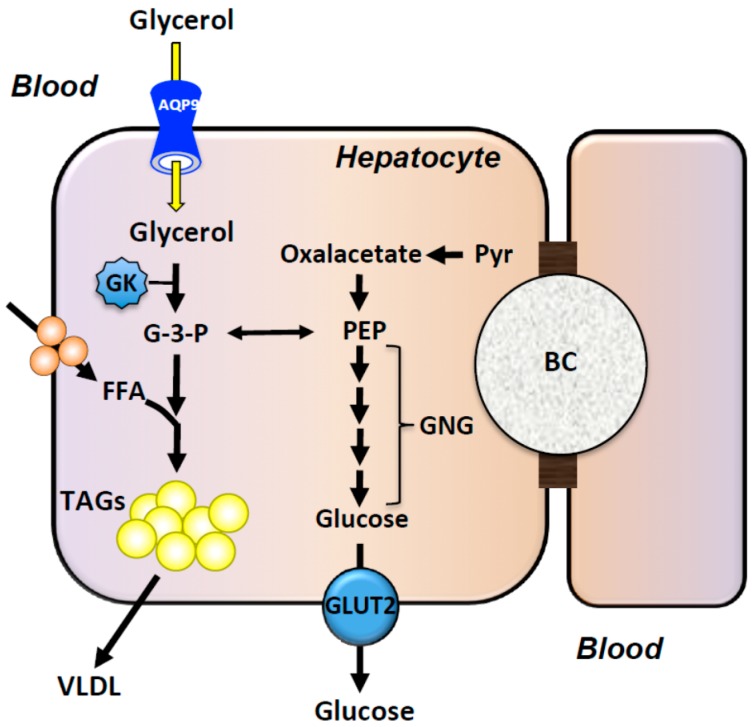
Proposed model of glycerol uptake by Aquaporin 9 (AQP9) in hepatocytes. Glycerol is imported from the sinusoidal blood through the membrane facilitated pathway created by AQP9. Once in the cell interior, glycerol kinase (GK) phosphorylates glycerol (G-3-P) to sustain gluconeogenesis (GNG) or triacylglycerols’ (TAGs) synthesis. BC, bile canaliculus; FFA, free fatty acids; GLUT2, glucose transporter 2; PEP, phosphoenolpyruvate; Pyr, pyruvate; VLDL, very-low-density lipoprotein.

Once imported into hepatocytes, glycerol is transformed in glycerol-3-phosphate to be used in triacylglycerols’ (TAGs) synthesis and as substrate for hepatic gluconeogenesis during fasting. Hepatocytes underlie most of the whole-body glycerol metabolism [36]. AQP9 is the principal facilitated pathway through which glycerol enters hepatocytes [31] and, at least in rodents, glycerol utilization is rate-limited by AQP9 facilitated import in hepatocytes in the first 24 h of starvation [30,31] (Figure 1). The interplay among glycemia, plasma insulin and hepatocyte AQP9 was recently modeled mathematically, using Hill and step functions, as an initial step in simulating the refilling/depletion of glycogen stores in the fed, fasted and re-fed states [37]. More recently, we devised a system of nonlinear first-order ordinary differential equations to set up a mathematical model of the hepatic glucose metabolism. A dataset of the time course of plasma glucose and insulin integrated with the hepatic glycogen content simulated the expression of AQP9 and glycerol permeability in mouse hepatocytes in various metabolic states (Gena et al. manuscript in preparation). While aiding the understanding of the role of liver AQP9 in rodent glycerol homeostasis, the devised mathematical approach may represent a fundamental step in predicting the function of the human liver as a module of a whole-body model of energy metabolism, both in health and disease.

The peroxisome proliferator-activated receptor α (PPARα) was suggested to be critical for the increased expression of AQP9 in male (but not female) mice during fasting [38]. The observation that hepatocyte AQP9 in male rats is down-regulated transcriptionally in response to agonists of PPARα suggests that, in fed conditions, activation of PPARα directs glycerol to triacylglycerols (TGAs) synthesis rather than into gluconeogenesis [39]. Disruption of the *Aqp9* gene in obese diabetic *db*/*db* mice allegedly diminishes glycemic concentrations [40]. In rodents, insulin downregulates hepatocyte *Aqp9* gene transcription by acting on an insulin response element (IRE) [41], which is consistent with AQP9 augmentations observed in animal models of insulin resistance [26]. Gender-related differences were seen in rats where 17β-estradiol prevented increased hepatic AQP9 expression and glycerol uptake during fasting [29] (for review, see [27]). Besides AQP9, human hepatocytes are reported to express three other aquaglyceroporins: AQP3, AQP7, and AQP10, although to low extents [42]. The expression and control of AQPs in the human liver seems to be distinct from the regulation that occurs in mice. In HepG2 cells, a human hepatoma cell line, AQP9 was found to be up-regulated by insulin through the phosphatidylinositol 3-kinase/protein kinase B/mammalian target of rapamycin (PI3K/Akt/mTOR) signaling cascade. On the other hand, the gene expression of AQP9 was reduced by leptin [43] and AMP protein kinase (AMPK), via forkhead box a2 (Fox a2) [44]. Variable results have been observed regarding the hepatic expression profile of AQP9 in obese subjects with type 2 diabetes mellitus (T2D) and obese subjects with no impairment of their glucose tolerance [43,45]. Like in rodents, human hepatocyte AQP9 undergoes sexual dimorphism. Obese women have lower liver permeability to glycerol compared to obese men, however, the expression levels of AQP9 did not significantly differ [42]. This may help explain why insulin resistance and the worrisome pathology Non-Alcoholic Fatty Liver Disease (NAFLD) display lower incidence in female than in male individuals.

Aquaglyceroporins have been associated with caveolins as integral membrane proteins implicated in maintaining metabolic and energy homeostasis [46]. Although considerable evidence exists suggesting relevance for AQPs in metabolism and energy balance [47,48], additional work will be necessary to fully clarify their regulation of metabolic homeostasis.

#### 2.2.2. Aquaporin 8 (AQP8) in Mitochondrial Ammonia Detoxification

AQP8 efficiently facilitates the membrane diffusional transport of ammonia in rat, mouse and human testis plasma membrane vesicles [20,49,50,51,52]. Moreover, mitochondrial AQP8 (mtAQP8) is able to markedly increase ammonia transport across inner mitochondrial membranes [53].

Ammonia generated from protein catabolism is mainly detoxified in hepatocytes through conversion to urea in the urea cycle, a pivotal process for preventing hyperammonemia and hepatic encephalopathy and implicating mitochondrial uptake of ammonia. No relevant role was found for hepatocyte mtAQP8 in whole mitochondrial water permeability (for review, see [19]), although an important role for mtAQP8 in ammonia detoxification via ureagenesis was suggested based on recent experimental data [22]. Basal and glucagon-induced ureagenesis from ammonia were significantly decreased in hepatocytes after mt*AQP8* knockdown [22]. On the contrary, mt*AQP8* silencing induced no considerable variation in ureagenesis when glutamine or alanine, two intramitochondrial nitrogen donors, were used [22]. Corroboration for an mtAQP8-facilitated ammonia transport to sustain urea cycle was compiled from in vivo works using a rodent model. In this model, glucagon-induced ureagenesis was associated with up-regulation of both hepatic mtAQP8 protein levels and diffusional ammonia permeability of inner mitochondrial membranes [22]. In addition, liver mtAQP8 was upregulated in rats with hypothyroidism, a condition characterized by increased hepatocyte urea synthesis [54]. Evidence is also available implying mtAQP8 in the pathogenesis of impaired hepatic ammonia detoxification in sepsis. Lipopolysaccharide treated rats displayed a down-regulation in hepatocyte mtAQP8 and mitochondrial ammonia diffusion associated with impaired basal and glucagon-stimulated synthesis of urea from ammonia [55].

AQP9 was reported to contribute to the exit of urea from mouse hepatocytes where an elusive urea transporter (UT)-like carrier may be more important in hepatic urea extrusion [32].

#### 2.2.3. Aquaporin 8 (AQP8) in the Hepatic Metabolism of Glycogen

AQP8 was also reported to be present in smooth endoplasmic reticulum (SER) membranes adjacent to glycogen granules of rat hepatocytes [9]. Hence, a role for AQP8 was suggested in maintaining the osmolality of cytoplasm during the synthesis and degradation of glycogen. Speculatively, AQP8 may facilitate the rapid flux of water between SER lumen and cytoplasm. Additional work is necessary to evaluate this hypothesis.

### 2.3. Roles in Primary Bile Formation and Secretion

**Figure 2:**
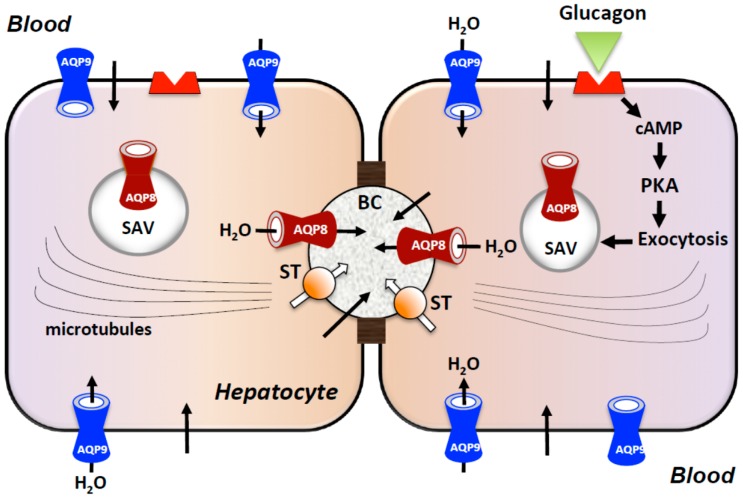
Working model of AQP-facilitated water movement in canalicular bile formation. AQP8 mediates osmotically-driven water secretion into the bile canaliculus (BC), whereas sinusoidal AQP9 contributes to the cellular uptake of water. The choleretic hormone glucagon, after binding to its receptor (in red), stimulates the microtubule-dependent canalicular targeting of AQP8-bearing vesicles located subapically (SAV, subapical vesicles). During the agonist-stimulated hepatocyte bile formation, the transcellular movement of water is coupled osmotically to the active transport of bile salts through pumps and exchangers. PKA, protein kinase A; ST, solute transporters. Black arrows indicate water transport; white arrows indicate solute transport.

Canalicular bile formation is an osmotic secretory process where water transport across hepatocytes plays a significant role. Osmotically active substances, mainly bile salts and other organic anions, are actively transported into bile canaliculi, resulting in the passive entry of water [16]. Thus, the biliary excretions of bile salts and organic anions are thought to be the main driving forces for water movement from the sinusoidal blood to the bile canaliculus (for review, see [56]). Osmotically-driven transepithelial water transport is largely transcellular via AQPs with minor paracellular contribution [5,56]. AQP8 is responsible for the rate-limiting water secretion at canalicular membranes during bile secretion [57,58,59], whereas AQP9 is involved by contributing to the sinusoidal uptake of water [5]. Moreover, choleretic hormones, such as glucagon and endothelins, increase canalicular AQP8 expression [11,60]. Altogether, these studies suggest AQP8 to underlie the mechanism through which water transport is coupled osmotically to active solutes during agonist-stimulated hepatocyte bile secretion (Figure 2).

### 2.4. Mitochondrial Aquaporin 8 (AQP8) and Reactive Oxygen Species (ROS) Release

Hepatic mitochondria are important sources for hydrogen peroxide generation. These reactive oxygen species (ROS) are normally released from hepatocyte mitochondria and then involved in signal transduction pathways [61]. AQP8 can work as a peroxiporin facilitating the transmembrane transport of H_2_O_2_ [62,63]. Recent studies in human hepatocarcinoma HepG2 cells indicate that mtAQP8 mediates mitochondrial H_2_O_2_ release [23]. Moreover, *AQP8*-knockdown caused ROS-induced mitochondrial depolarization via the mitochondrial permeability transition mechanism, and finally necrotic death [23,64]. This may represent some potential towards conceiving therapeutic strategies against hepatoma cells. In non-hepatic cells, AQP8 has also been found to modulate NAD(P)H oxidases (Nox)-produced H_2_O_2_ transport through plasma membranes [63]. Whether hepatocyte canalicular AQP8 is involved in such a mechanism demands further investigation.

### 2.5. Hepatocyte Aquaporins in Fatty Liver Disease, Obesity and Diabetes Mellitus

**Figure 3:**
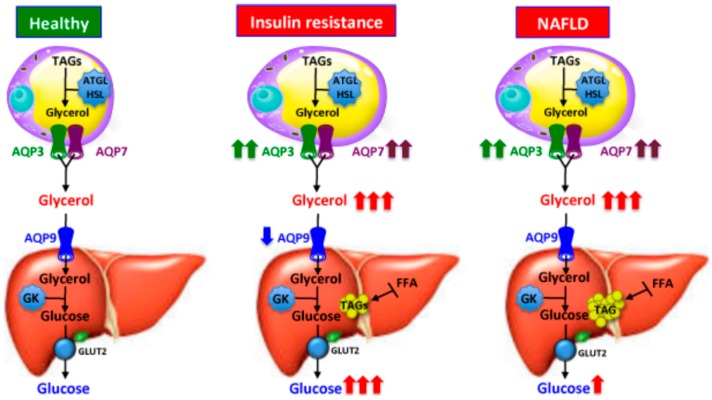
Working model for the function of aquaglyceroporins in the onset of insulin resistance and Non-Alcoholic Fatty Liver Disease (NAFLD) in humans. Insulin and leptin are regulatory factors for the expression of adipocyte AQP3 and AQP7 and hepatocyte AQP9. Circulating insulin and leptin levels vary in accordance to the metabolic state and adiposity, respectively. Hence, the expression of aquaglyceroporins in fat tissue and hepatocytes augments or diminishes in function of the nutritional needs and excess adipose mass. In the setting of obesity-associated insulin resistance and NAFLD, despite the hyperleptinemia, adipocyte AQP3 and AQP7 undergo overexpression. This leads to the increase of glycerol output from fat cells and glycerol use for hepatic gluconeogenesis and lipid synthesis. The reduced levels of AQP9 and glycerol permeability in the liver of obese subjects with insulin resistance is speculated to be a counteracting mechanism to prevent a further aggravation in liver steatosis and hyperglycemia. ATGL, adipose tissue triacylglycerol lipase; GLUT2, Gcose transporter, type 2; HSL, hormone-sensitive lipase; FFA, free fatty acids; GK, glycerol kinase; TAGs, triacylglycerols.

Non-Alcoholic Fatty Liver Disease (NAFLD), a worldwide health problem characterized by ectopic accumulation of TAG in the liver [65], is a frequent form of metabolic syndrome often associated with obesity and diabetes and connected to insulin resistance. NAFLD pathogenesis has been heavily investigated, especially regarding complex systems resulting in excessive TAGs’ accumulation in liver parenchyma [66]. A role for AQP9 in regulating hepatic TAG synthesis in NAFLD was suggested when it was shown that both liver AQP9 and hepatocyte glycerol permeability are diminished in mouse models of NAFLD [67] and in subjects with obesity, insulin-resistance and NAFLD [42]. AQP9 down-regulation and reduction in hepatic glycerol permeability in insulin-resistant conditions were interpreted in a way whereby the hepatocytes counteract further fat accumulation within its parenchyma and diminish hepatic gluconeogenesis during NAFLD. In any case, this scenario should be contextualized within the patho-physiological pattern and gender of the investigated animal and human specimens because the extent of AQP9 protein and liver import of glycerol had a distinct profile of control in *n*3-PUFA (ω3 polyunsaturated fatty acids)-depleted female rats [68], a model of metabolic syndrome having several features of the disease also including liver steatosis, and in rats [69] and mice [70] fed a high-fat diet. It is possible that AQP9 is increased in the early onset of steatosis and reduced at a later stage of the pathology, when consistent and excessive fat accumulation has occurred. Additional work needs to be carried out before exhaustively assessing both the patho-physiological meaning and control (i.e., the sex-specific dimorphism showed by morbid individuals) of AQP9 in a multifactorial pathology such as NAFLD. Figure 3 reports the interplay between liver AQP9 and adipose AQP3 and AQP7 in NAFLD states associated with obesity and T2D. The potential selective modulation of aquaglyceroporins and caveolins in liver and other metabolic organs in the therapy of NAFLD and other severe metabolic disease would also be worth further investigation [46].

### 2.6. Relevance in Bile Secretory Disorders

Cholestasis, functionally defined as an impairment of bile flow, is associated with several liver disorders. Chronic cholestasis causes liver injury, ultimately leading to cirrhosis and liver failure [71]. Defective canalicular AQP expression may lead to alterations of normal bile physiology. In fact, down-regulated canalicular AQP8 expression is present in experimental models of cholestasis [72,73,74,75] and could result from proteosomal and lysosomal proteolysis. The fact that a reduced sinusoidal water uptake may represent another contributing factor in obstructive cholestasis is suggested by the finding that the expression of hepatocyte AQP9 was down-regulated in obstructive cholestasis [76]. These findings support the notion that AQPs are involved in the development of bile secretory dysfunction.

The gene transfer of human *AQP1* (*hAQP1*) via the adenoviral vector *AdhAQP1* has been successfully used to restore normal salivary flow to the irradiated hypofunctional salivary gland of experimental animals [77] and humans [78]. We showed that *AdhAQP1*, when administered to estrogen-induced cholestatic rats by retrograde bile ductal infusion, increased bile flow [79]. *AdhAQP1* induced hepatocyte canalicular hAQP1 expression as well as an increase in canalicular osmotic water permeability and in the choleretic efficiency of endogenous bile salts (i.e., volume of bile/μmol of excreted bile salt) [79]. This suggests that bile flow was somewhat improved by hAQP1-mediated canalicular water transport [79]. An unanticipated result in hAQP1-transduced cholestatic animals was the noteworthy improvement in the biliary bile salt output caused by increased activity of the canalicular bile salt transporter Bsep/ABCB11 [80]. Our data suggested that hepatic adenoviral transfer of hAQP1 gene to estrogen-induced cholestatic rats improves biliary failure by increasing both biliary excretion and choleretic efficiency of bile salts [79,80].

### 2.7. Involvement of Hepatocyte Aquaporins in Other Diseases

Using *Aqp11* gene knockout mice, Ishibashi and coworkers suggested a role for hepatocyte AQP11 in cystic liver disease [81]. Later on, using mice carrying a liver-specific ablation of AQP11, other authors showed rapid vacuolization of periportal hepatocytes RER after amino acid feeding [82]. AQP9 was reported to be involved in the hepatocellular carcinoma [83]. A recent study suggested no general contribution for AQP9 to carcinogenesis [84], an interpretation that is in agreement with AQP9 being down-regulated in human hepatocellular carcinoma and its over-expression being allegedly involved in the suppression of hepatoma cell invasion, through the inhibition of the epithelial-to-mesenchymal transition [85].

### 2.8. Potential Pharmacological and Gene Transfer Applications

Therapeutic modulation of AQP expression or function is one of the most challenging topics in the field of AQPs. Attempts in designing or finding selective and effective small molecule blockers of AQP have yielded only a few hits. Difficulties mostly arise from the high copy number of AQP in the plasma membranes, and the spatial restrictions characterizing their protein structure (for review, see [86]). Regarding liver AQP9, inhibition of AQP9-facilitated glycerol import by hepatocytes [31] may be effective in the prevention of liver steatosis and some of its severe consequences, such as steatohepatitis and cirrhosis [42], or in the control of gluconeogenesis from glycerol in T2D. Small compounds efficiently and selectively inhibiting the AQP9-mediated glycerol transport in primary hepatocytes were described by Jelen and coworkers [30]. Structural derivatives of these blockers with low micromolar half maximal inhibitory concentration (IC_50_) values in AQP9-expressing Chinese hamster ovary cells were recently identified and the related putative intracellular binding sites localized by molecular dynamic simulation and molecular docking [87]. Additional work is now needed to synthesize structural analogues of these compounds with sufficient water solubility to be tested in vivo.

Hepatic adenoviral transfer of *AQP1* gene, as mentioned above, may be a valuable novel treatment for some liver cholestatic disorders [79,80].

## 3. Aquaporins in Sertoli Cells: Expression, Physiology and Potential Roles in Male Reproductive Potential

### 3.1. The Sertoli Cell: A Brief Overview

The testes are functionally compartmentalized organs, divided into seminiferous tubules and interstitial space, where spermatogenesis and testosterone biosynthesis take place, respectively. Sertoli cells (SCs) are the somatic cellular component which is essential for testis formation and are responsible for the compartmentalization of this organ and the support of spermatogenesis [88]. Adjacent SCs are connected by tight junctions, establishing the Sertoli/blood-testis barrier (BTB), which allows them to create a protected environment within the seminiferous tubules [88]. These somatic cells, also called testicular “nurse cells”, play five essential roles that allow the occurrence of the spermatogenic event: (1) formation of the BTB; (2) nourishment and structural support to the developing germ cells; (3) elimination of defective germ cells; (4) production of fluid tubular seminiferous and other factors of regulation; (5) creation of an immune-privileged environment [87,89]. SCs are cells of large dimensions, with columnar shape, which are adherent to the basal lamina. They extend from the base of the seminiferous tubule to its lumen, where spermatozoa are released. These cells exhibit several particular features including large quantities of mitochondria, lipid droplets, glycogen particles and specific hormone receptors [90,91,92]. In fact, hormones are crucial regulatory factors for the functioning of the SCs [93,94], particularly gonadotrophins (mainly follicle stimulating hormone—FSH), thyroid hormones, sex steroid hormones and insulin. For instance, compelling evidence has shown that FSH is essential for the establishment of male reproductive potential and particularly for SC physiology. SCs also possess a large variety of membrane transport proteins in their membranes, allowing them to control the seminiferous fluid composition and pH [95,96,97,98].

### 3.2. Testicular Metabolic Cooperation between Sertoli: Germ Cells: A Selective Process of Nutrients and Fluids

The metabolism of SCs is a central player in the normal occurrence of spermatogenesis and presents distinctive characteristics. As previously stated, SCs regulate the selective passage of substances from the interstitial fluid to the adluminal compartment, which is filled with the seminiferous tubular fluid (STF) [99]. Among these substances, we must emphasize the importance of energy metabolites. It is known that SCs produce large amounts of lactate, mostly by the conversion of glucose via glycolysis [100]. Notably, the SC-produced lactate is then used by germ cells under development that are incapable of using glucose as an energetic source. In fact, lactate is the ideal metabolic substrate for developing germ cells [101] and acts as an anti-apoptotic factor in these cells [102], through mechanisms that remain a matter of debate. In sum, SCs present a Warburg-like glucose metabolism, similar to what is observed in cancer cells [103], favoring the fermentative (rather than oxidative) metabolism of glucose, despite being a less effective pathway in terms of ATP production [104].

**Figure 4:**
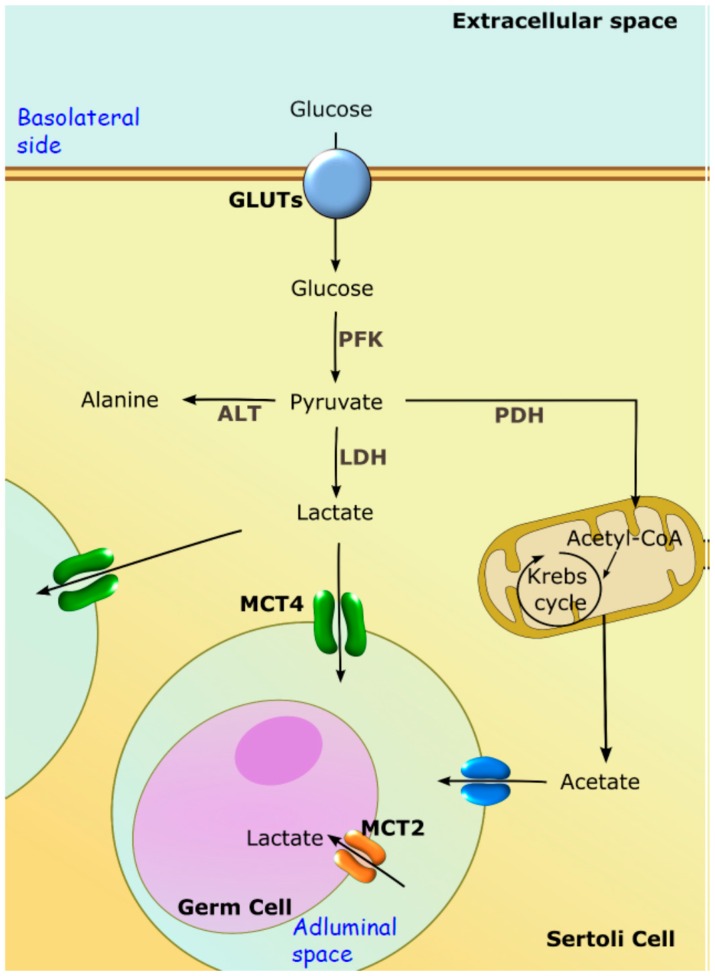
Schematic diagram of the metabolic cooperation between Sertoli cells (SCs) and developing germ cells. The glucose taken up from the extracellular space enters SCs through glucose transporters (GLUTs). The glucose is then converted to pyruvate through glycolysis. Pyruvate can follow multiple pathways. However, in these cells, most glucose is used to produce, via lactate by lactate dehydrogenase (LDH). Lactate is then transported out of SCs by specific monocarboxylate transporters (MCT4). Germ cells take up the lactate produced by SCs through MCT2. Of note, as happens in other cells, pyruvate can also be converted into alanine (by alanine aminotransferase—ALT) or transported to mitochondria, forming acetyl-CoA (by pyruvate dehydrogenase—PDH). Acetyl-CoA is then converted into acetate that may be used by germ cells for lipid synthesis. PFK, phosphofructokinase.

In SCs, glucose crosses the plasma membrane through specific Glucose transporters (GLUTs). Up until now, four GLUTs isoforms have been consistently reported in the plasma membrane of SCs, namely GLUT1, GLUT2, GLUT3 and GLUT4 [105,106]. After glucose crosses the plasma membrane, it is converted into pyruvate via glycolysis. Most of the pyruvate originated from glycolysis is converted by lactate dehydrogenase (LDH) into lactate, which is the preferred metabolic substrate of developing germ cells [88,107]. The lactate produced is then released to the STF by specific membrane transporters present on SCs. So far, only monocarboxylate transporters (MCTs), particularly MCT1 and MCT4, have been implicated in the export of lactate by these cells. The MCTs are responsible for the export of lactate to the intratubular fluid, where it may be used by developing germ cells [107,108] (Figure 4). Besides being the main energy source for ATP production by developing germ cells [88,93], lactate is also thought to be essential for the control of the STF pH, since lactate transport is coupled with H^+^ and thus, a shifting of pH can be expected.

Recently, it has been described that, in addition to lactate, SCs also release high amounts of acetate to the extracellular environment [109]. However, the exit route from SCs or the role of acetate in spermatogenesis is not yet fully understood. The authors suggested that it may be useful to maintain the elevated rate of lipid synthesis that is necessary for germ cell division, being an intermediate for the synthesis of cholesterol and fatty acids [110] and thus, pivotal for the formation of membranes (Figure 4). Hence, spermatogenesis is completely dependent on the metabolic cooperation between SCs and developing germ cells [92]. In fact, compelling evidence suggests that alterations in these metabolic processes may result in deleterious outcomes for the reproductive potential of males or be involved in subfertility, or even infertility, induced by several diseases [111]. Cells from the germ line are completely dependent on carbohydrate metabolism (both on the aerobic and anaerobic pathways) [112]. On the other hand, sperm cells, which lie in the adluminal compartment, exhibit a great metabolic flexibility, using different metabolic pathways for energy production [101] while spermatocytes exclusively depend on lactate supply by SCs [112]. Lactate and pyruvate are known to be essential to germ cells at later developmental stages for energy production [113]. The fact that the testes are oxygen-deprived organs [114] can explain why germ cells may use these distinctive metabolic pathways to obtain energy in their different stages of development. Indeed, several studies have demonstrated that spermatogenesis is completely dependent on the metabolic cooperation established between testicular cells and on the production of lactate by SCs. This cooperation is also known to be controlled by fluid composition and pH. In fact, and although this is a matter that has been so far overlooked, some very fine reports have shown the relevance of fluids for this process in the last few years.

### 3.3. Expression and Subcellular Localization of Aquaporins in Sertoli Cells

Fluid absorption and secretion are vital processes that occur in the male reproductive tract [115]. Water movements are essential in determining the composition of the luminal fluids that fill the testicular ducts and for providing a means of transport to the spermatozoa into the epididymal ducts. Therefore, it is not surprising that AQPs have emerged as pivotal players in those mechanisms and that the expression of various AQPs has been described in several testicular cells [116,117,118,119,120], which is consistent with the occurrence of water-dependent fluid movement in testes.

As said, SCs are the main mechanism responsible for the secretion of the fluid that fills the seminiferous tubules [121] and this mechanism is expected to rely on the participation of the various AQPs isoforms already described in these cells. While the presence of the majority of the known AQP isoforms has been reported to be present in the testis and/or in the ducts of the male reproductive tract [97,122], only a few of those isoforms (AQP0, AQP4, AQP8 and AQP9) are known to be expressed in the SCs, although the data available is not always consistent and very few studies addressed the functional aspects of these channels (Table 2) [97,123].

AQP0 expression in the seminiferous epithelium seems to be restricted to SCs. In these cells, AQP0 is expressed in a specific semicircular pattern, which changes in the different stages of spermatogenesis [119]. Because AQP0 is expressed in SCs at stages VI–VIII of the spermatogenic cycle, it has been suggested that this AQP is implicated in the transport of water from the interstitial space into the lumen of the seminiferous tubules during those specific periods, which correlate with the release of the elongating spermatids into the lumen of the seminiferous tubule. Thus, AQP0 seems to promote the movement of spermatozoa into the epididymis by facilitating the transport of water into the lumen of the seminiferous tubules. Nevertheless, further studies are needed to unveil the functional relevance of AQP0 for the movement of spermatozoa into the epididymis, since it is a pivotal event for the reproductive success of the males.

Regarding AQP4, limited data is available concerning its presence in the male reproductive tract. Recent data described its presence in rat SCs [124]. Actually, AQP4 is one of the more abundantly expressed AQPs in the equine testis [125]. Yet, no data is available on the function of this AQP in the various testicular cells or specifically in SCs. Nonetheless, as AQP4 is abundantly expressed in cells that support the blood-brain barrier [126]—a structure similar to the BTB [127], playing a central role in water balance and ion homeostasis in the brain—it has been suggested to serve an analogous function in the testis and particularly in SCs (Table 2).

AQP8 was also identified in rat SCs [118,128]. Interestingly, in the rat seminiferous epithelium, the expression of AQP8 was exclusively localized on SCs [118], where it is found homogeneously in every tubule, which is consistent with a constitutive expression of this AQP in SCs [7]. However, in contrast with the abundance of AQP8 in rat testis, AQP8 seems to be absent in the human testis [129]. Based on the role that this AQP plays in the cells where it is expressed, the presence of AQP8 in SCs suggests that it may be involved in the transport of water from the interstitial space into the lumen of the tubules. This movement of water seems to occur along an osmotic gradient, originated by the action of several other membrane transporters [130] (particularly the Na^+^/K^+^-pump, which has been co-localized with AQP8 on the adluminal portion plasma membranes of SCs) [118]. The wide presence of this AQP in the adluminal plasma membrane of SCs at all stages of the cycle of the seminiferous epithelium leads to the suggestion that it may cooperate with other AQP isoforms in the seminiferous epithelium [118,128]. It has been proposed that the transition of water into the lumen of the seminiferous tubules may be enhanced by the cooperation of other AQP isoforms which co-localize with AQP8 in SCs (such as AQP0). However, somewhat unexpectedly, *Aqp8* null mice exhibited only mild phenotype differences on the reproductive organs when compared with the wild type and *Aqp8*^+/−^ heterozygous mice. Moreover, even though testis weight and size in *Aqp8*^−/−^ mice were increased, no significant alteration on sperm parameters or impaired fertility were described in these rats [131].

Lastly, the presence of AQP9 has been detected at high levels throughout the male reproductive tract, even though AQP9 null mice are fertile [40]. The data available suggests that the expression of AQP9 is cell-specific in testes [118], with its presence being reported in rodent germ cells (particularly spermatocytes at early developmental stages) [120] and in the plasma and intracellular membranes of interstitial Leydig cells [40,118]. AQP9 expression has also been reported in SCs [120,132], suggesting that this AQP may play an essential role in the transport of water and/or non-charged solutes in all these testicular cells [133], similarly to what happens in astrocytes, the key components of the blood-brain barrier.

### 3.4. Aquaporins Functionality in Testis and Their Possible Relevance for Sertoli Cell Metabolism

**Figure 5:**
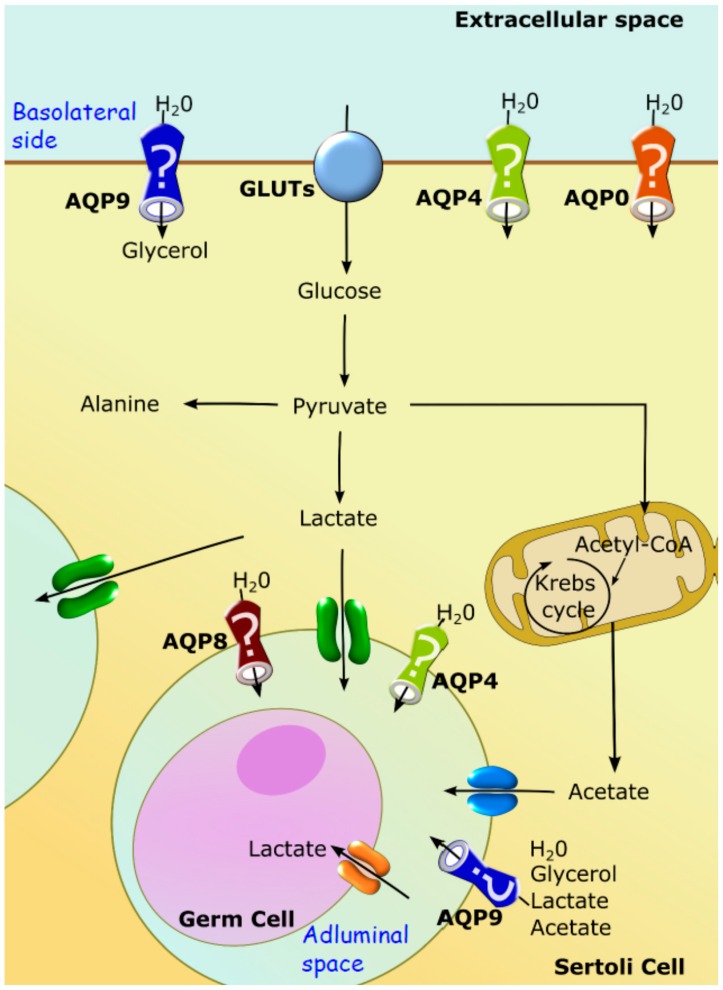
Schematic diagram of the possible subcellular localizations of AQPs in Sertoli cells and their possible link to metabolism. SCs express multiple AQP homologues, AQP0, AQP4, AQP8 and AQP9. The basolateral plasma membrane is believed to contain AQP0, AQP4 and AQP9. Basolateral AQP9 may be relevant for testicular metabolic cooperation. The SC adluminal plasma membrane contains AQP4, AQP8 and AQP9. Adluminal AQP9 may mediate the extrusion of metabolic intermediates such as lactate, acetate and glycerol (in addition to water). The functional significance of adluminal AQP4, an AQP highly permeable to water, and AQP8, a homologue conducting water and some other molecules, remains elusive.

While it has been described that orthodox AQPs, such as AQP0 and AQP4, are mostly permeable to water, being responsible for the establishment of cellular and/or transcellular fluxes, other non-conventional AQP isoforms are known to be permeable to additional non-charged solutes, and play distinct roles in the physiology of the cells and tissues where they are expressed [134]. In the testicular tissue, and particularly in the seminiferous tubules, it has been suggested that both AQP0 and AQP4 should participate in water balance and ion homeostasis, and that the presence of these transporters in SCs may be part of a mechanism that helps to create a route that facilitates the transepithelial movement of water in the seminiferous tubules [124]. As previously mentioned, these water movements are essential for providing a means of transport to spermatozoa release into the lumen of the tubules into the excurrent duct system and also for controlling the composition of the luminal fluid that fills the seminiferous tubules (Figure 5).

Regarding AQP8, it has been proposed that this AQP could be involved in the transcellular movement of water from the interstitial space into the lumen of the seminiferous tubules [123]. The finding that AQP0 is expressed at high levels in these cells during specific stages of the spermatogenic cycle suggests that it may assist AQP8 in its function. As previously mentioned, AQP8 was described as being present in the adluminal plasma membrane of SCs [118] and should be responsible for the efflux of water from the SCs, while AQP0 might be involved in its uptake from the interstitial fluid (Figure 5). Therefore, the presence of these two AQPs at the specific time point of the cycle may facilitate the transport of water into the lumen and hence the movement of the spermatozoa out of the seminiferous tubules.

Contrastingly, AQP9 is an aquaglyceroporin that might be involved in distinct events in the testicular tissue other than the movement of water. As reported before, AQP9 is permeable to water, urea, glycerol and monocarboxylic acids, namely lactic acid and acetic acid, while being impermeable to cyclic sugars (e.g., d-glucose) [135]. The expression of AQP9 in the seminiferous epithelium [40,132] and its selective permeability lead to the suggestion that this AQP might be involved in the transport of non-charged energy metabolites in the BTB. Similarly to what happens in the blood-brain barrier (and particularly in astrocytes) [135], the presence of AQP9 in SCs supports a role in testicular metabolism as a glycerol and monocarboxylic acid channel. As discussed before, although glucose is the major source of energy for most testicular cells, developing germ cells that lie beyond the BTB depend on lactate as an energetic substrate [111]. The presence of AQP9 in SCs suggests that it may facilitate the diffusion of lactate to the intratubular fluid in conjunction with the MCTs already identified in these cells, particularly MCT4, which is reported to be highly expressed in SCs [92]. We may also hypothesize that glycerol can diffuse through AQP9 channels into SCs, although the current knowledge concerning the metabolism of this polyol in the testicular cells is scarce. Still, it has been described that an increase in the testicular levels of glycerol (by exogenous administration) transiently compromises spermatogenesis, also leading to disruption of the BTB [136]. Taking this into account, AQP9 may play a crucial role in the success of spermatogenesis, particularly in pathological conditions associated with increased plasma glycerol levels (e.g., obesity, diabetes mellitus). Taken together, the data available raises the hypothesis that AQP9 in SCs may play a role in testicular energy metabolism and metabolic cooperation as a lactate (glycerol) channel. This hypothesis still waits functional validation and the molecular mechanisms by which AQP9 may control the metabolic cooperation between SC:germ cells are still unknown.

Therefore, there is still much to unveil concerning the function of AQPs in the mammalian cells of the male reproductive tract, particularly in SCs, and their role in the processes that define successful functional sperm production. However, there are recent compelling evidences suggesting that AQPs are crucial for normal spermatogenesis and, thus, for overall male reproductive health. Nevertheless, their role and relevance for male fertility remain a matter of intense discussion. It is expected that dysregulation of AQPs function may be involved in the subfertility, or even infertility, induced by several diseases in males. This is a research scope that has been so far overlooked. The role that AQPs play in male fertility deserves a special attention from researchers in the years to come, and may represent an exciting field, unveiling novel mechanisms to control male fertility.

## 4. Conclusions and Future Perspectives

Investigation on hepatocyte and Sertoli cell AQPs is highly instructive, providing useful information about critical functions such as energy balance, ammonia detoxification, ureagenesis and mitochondrial ROS generation and signaling, without neglecting critical functions such as the modulation of insulin secretion, and spermatogenesis. Nevertheless, further work is necessary to fully clarify the mechanisms through which the involved AQPs are controlled in their expression and function, especially when trying to translate the information obtained with cellular and animal models to human beings. Additional functions for AQPs are sure to be unraveled in the next years. The patho-physiological implication of AQPs in the onset of human pathologies is an exciting new field of research, with putative important key diagnostic, biotechnological and pharmacological implications. Identification of compounds able to inhibit AQPs is among the top priorities in the field.

## Figures and Tables

**Table 1:** Subcellular localization and suggested significance of hepatocyte aquaporins (AQPs) in health and disease.

Aquaporin	Subcellular Location	Suggested Functional Involvement	Suggested Clinical Relevance
AQP8	AM; SAV; SER; IMM	Secretion of canalicular bile water; preservation of cytoplasm osmolarity during glycogen synthesis and degradation; mitochondrial ammonia detoxification and ureagenesis; mitochondrial H_2_O_2_ release	Cholestasis
AQP9	BLM	Uptake of glycerol during starvation; import of water from sinusoidal blood; urea extrusion	Cholestasis; T2D; NAFLD; Hepatocellular carcinoma
AQP11	RER	RER homeostasis; liver regeneration	Foam-like hepatocyte

AM, apical plasma membrane; BLM, basolateral plasma membrane; IMM, inner mitochondrial membrane; NAFLD, Non-Alcoholic Fatty Liver Disease; RER, rough endoplasmic reticulum; SER, smooth endoplasmic reticulum; SAV, subapical membrane vesicles; T2D, type 2 diabetes mellitus.

**Table 2:** Sertoli cell distribution and possible functions of mammalian Aquaporins.

Aquaporin	Testicular Distribution	Suggested Function
AQP0	Sertoli cells and Leydig cells	Establishment of an adequate luminal environment in the seminiferous tubule; Transport of water from interstitial space into the lumen of the seminiferous tubule, in order to promote the movement of spermatozoa into the epididymal ducts
AQP4	Sertoli cells	Regulation of extracellular space volume, potassium buffering, fluid circulation and reabsorption
AQP8	Sertoli cells and germ cells	Formation of the seminiferous tubular fluid
AQP9	Sertoli cells, Leydig cells, spermatocytes, efferent ducts, epididymis	Transport of water and non-charged solutes in Leydig cells; Formation of the seminiferous luminal fluid
